# Simultaneous Detection of 14 Microcystin Congeners from Tissue Samples Using UPLC- ESI-MS/MS and Two Different Deuterated Synthetic Microcystins as Internal Standards

**DOI:** 10.3390/toxins11070388

**Published:** 2019-07-02

**Authors:** Stefan Altaner, Jonathan Puddick, Valerie Fessard, Daniel Feurstein, Ivan Zemskov, Valentin Wittmann, Daniel R. Dietrich

**Affiliations:** 1 Human and Environmental Toxicology, University of Konstanz, 78457 Konstanz, Germany; 2 Cawthron Institute, 7010 Nelson, New Zealand; 3 Toxicology of Contaminants Unit, French Agency for Food, Environmental and Occupational Health and Safety, ANSES, 35306 Fougères, France; 4 Dr. Feurstein Medical Hemp GmbH, HANAFSAN, Hauptstr. 19A, 6840 Götzis, Austria; 5 Organic and Bioorganic Chemistry, University of Konstanz, 78457 Konstanz, Germany

**Keywords:** cyanobacterial toxin, deuterated MC standards, microcystin, blood, liver tissue, UPLC-MS/MS, quantification

## Abstract

Cyanobacterial microcystins (MCs), potent serine/threonine-phosphatase inhibitors, pose an increasing threat to humans. Current detection methods are optimised for water matrices with only a few MC congeners simultaneously detected. However, as MC congeners are known to differ in their toxicity, methods are needed that simultaneously quantify the congeners present, thus allowing for summary hazard and risk assessment. Moreover, detection of MCs should be expanded to complex matrices, e.g., blood and tissue samples, to verify in situ MC concentrations, thus providing for improved exposure assessment and hazard interpretation. To achieve this, we applied two synthetic deuterated MC standards and optimised the tissue extraction protocol for the simultaneous detection of 14 MC congeners in a single ultra performance liquid chromatography-tandem mass spectrometry (UPLC-MS/MS) run. This procedure was validated using plasma and liver homogenates of mice (male and female) spiked with deuterated MC standards. For proof of concept, tissue and plasma samples from mice i.p. injected with MC-LR and MC-LF were analysed. While MC-LF was detected in all tissue samples of both sexes, detection of MC-LR was restricted to liver samples of male mice, suggesting different toxicokinetics in males, e.g., transport, conjugation or protein binding. Thus, deconjugation/-proteinisation steps should be employed to improve detection of bound MC.

## 1. Introduction

**Figure 1:**
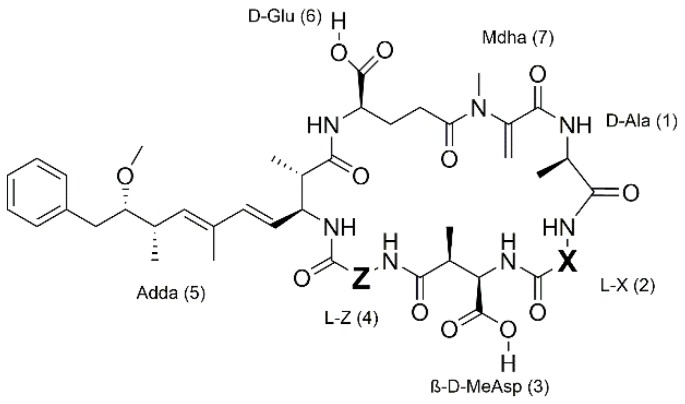
General structure of microcystins where X and Z are variable l-amino acid positions. β-d-MeAsp is erythro-β-d-methylaspartate; Mdha, N-methyldehydroalanine, Adda, (2S,3S,8S,9S,4E,6E)-3-amino-9-methoxy-2,6,8-trimethyl-10-phenyl-4,6-decadienoic acid.

In recent years, reports of cyanobacterial blooms have increased [1]. Water bodies experiencing cyanobacterial bloom formation often additionally contain toxins, like microcystins (MCs), which can pose a serious health threat to humans. This was demonstrated in Caruaru in 1996, when 76 patients died because of the use of contaminated water for hemodialysis [2]. MCs are cyclic heptapeptides, comprised of rare and unique l- and d-amino acids and two variable l-amino acid positions (X and Z, Figure 1), which are also used for nomenclature, e.g., MC-LR has l-leucine in the X position and l-arginine in the Z position. Along with the variable amino acids, methylations and demethylations at other positions have led to more than 200 congeners being identified to date [3].

Differential transport of MC congeners by organic anion transporting polypeptides (OATPs) has been shown to result in different cell and tissue loads [4,5]. Additionally, export of MC is still under research, although recent data suggest export of MC by MRP2 in an MC congener-dependent fashion as observed earlier for OATPs [6,7]. As the liver displays high expression of OATPs and MC exposure mainly occurs via the oral route, the liver is generally accepted to be the main target organ. In the target cells, MC is either freely available (unbound) or covalently bound to serine/threonine-protein phosphatases (PPPs), conjugated to glutathione [8] or other cysteine-containing polypeptides and proteins. The irreversible inhibition of PPPs by MCs, and consequently the downstream protein-hyperphosphorylation, is currently assumed to be the predominant mechanism underlying MC cytotoxicity. Most members of the PPP-family are known to be affected by MCs [9], albeit with different susceptibilities [10]. Indeed, structural features of MC congeners appear to have a major impact on the “tightness” of MC binding to the catalytic subunit of PPP [11,12], thus resulting in the observed differing PPP inhibition capacities. Thus, information as to whether a specific MC congener was involved in a given toxicity observed, and at what tissue concentration adverse effects manifest, is crucial for a better understanding of MC congener differences in exerting overt toxicities and consequently for better delineating risk for human health.

MC detection is generally carried out via ELISA or chromatographic methods coupled to mass-spectrometric analysis (LC-MS) [13,14]. ELISA methods may differ in the antibodies used, and thus their capability to detect the majority of MC congeners present in a given sample. Indeed, while antibodies raised against the arginine epitope in MC-LR primarily recognise arginine containing MC congeners [15], antibodies raised against Adda potentially recognise 85% of the known congeners [16]. Similarly, the MS-based MMPB method which detects the oxidation product MMPB (2-methyl-3-methoxy-4-phenylbutyric acid) of Adda [17,18], therefore showing similar coverage (86%). As with the ELISA, only the sum of all MC and not specific MC congeners can be quantified. An advantage of the MS-based MMPB method, however, is that protein-bound MC can be detected as both bound-MC and free MC are oxidised [18], albeit whether bound-MC and free MC oxidation occurs with the same efficiency is currently under debate [19].

UPLC-MS/MS-based methods provide the possibility of simultaneous detection of different congeners in various sample types [20,21,22,23,24,25]. However, quantitation from complex matrices like blood or tissue homogenate is difficult due to matrix residues which may influence the signal (suppression or enhancement), thus leading to under- or over-estimation of the true amount of MC present. This could be alleviated with internal standards (ISs) that behave similarly to the MC congeners of interest analysed. Unfortunately, appropriate internal standards are still missing. Quantitation from various samples using ISs has been attempted previously [20,26,27]. However, in these approaches, either nodularin or thiolised MC-LR/-RR at the Mdha moiety was employed. Obviously all three ISs used in the latter approaches will differ in their behaviour during sample extraction, elution and analysis. In contrast, incorporation of stable isotopes is the best option for the generation of an IS. The latter ensures that during laboratory handling and analysis, a stable isotope-labelled compound has near-identical behaviour and is subject to the same matrix effects as the actual analyte in question.

Detection of analytes from tissue is often complicated due to the need for tissue extraction, and thus tissue specific matrix effects. Generally, extraction methods are optimised for a given sample type and for a single or a small number of MC congeners, primarily representing rather hydrophilic congeners, e.g., MC-RR, -LR and -YR. However, in view of the fact that several different MC congeners co-occur in a given cyanobacterial bloom [28,29], including the highly toxic MC-LF [30], the present work aimed to establish a quantitation method for a wider range of congeners in complex matrices allowing the analysis of congener specific organ distribution. Thus, deuterated internal standards (D_7_-MC-LR and D_5_-MC-LF) were de novo synthesised, and present protocols for MC sample extraction were optimised and integrated into a UPLC-MS/MS-based analytical procedure. For proof of concept, tissue and plasma samples from MC-LR and -LF exposed mice were analysed.

## 2. Results

### 2.1. Method Establishment and Optimisation

**Figure 2:**
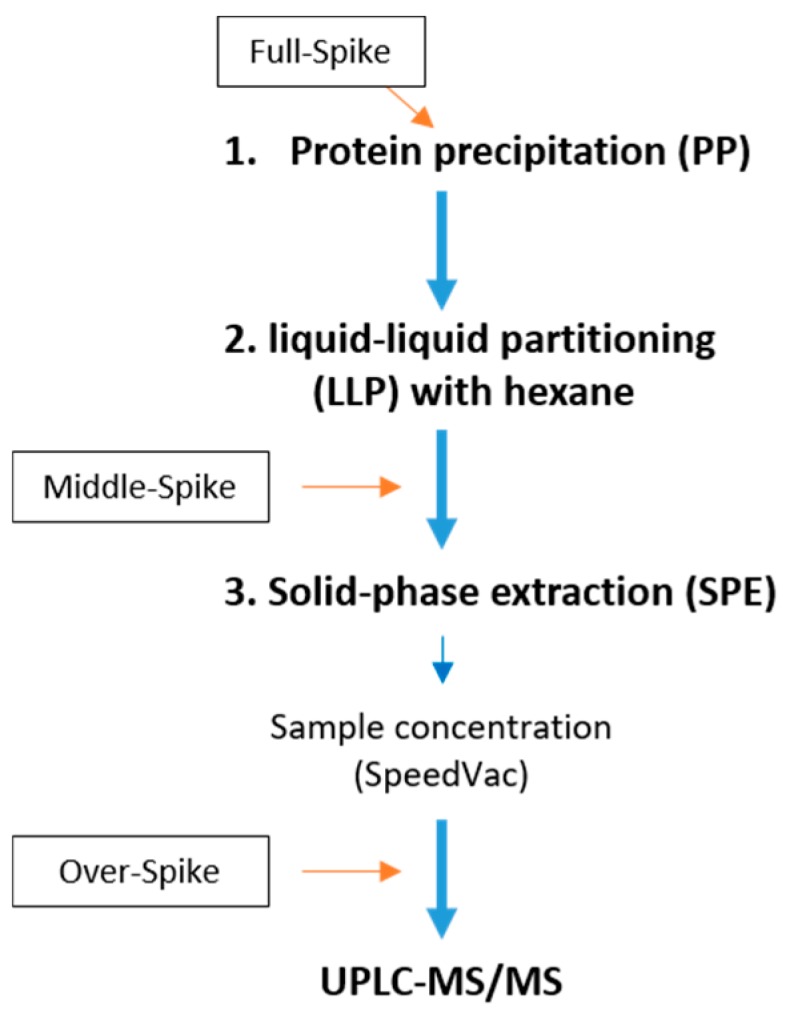
Scheme of the extraction highlighting different time points for spiking with MC congeners.

MC-spiked serum samples were extracted using a previously published procedure for detection via ELISA [31] and subsequently analysed using a previously established UPLC-MS/MS method optimised for MC in cyanobacterial culture extracts [32]. The extraction method (Figure 2) consisted of three times protein precipitation (PP), followed by three times liquid-liquid partitioning (LLP) with n-hexane and subsequent solid-phase extraction (SPE). In the process, signal enhancement was observed for all MC congeners tested (data not shown), presumably due to carry-over of the previous sample as signal enhancement progressively increased with every additional injection. Therefore, a slower gradient with a prolonged washout phase (see Section 4.4) was used during UPLC, leading to stable detection of the MC congeners tested (comparable areas under the curve for multiple subsequent analyses of the same MC congener).

To determine whether and during which extraction steps potential loss of MC congeners would occur, MC spiking was introduced at different points of the extraction procedure (Figure 2) and the respective losses, i.e., recoveries of spiked MC, determined at each step using a backward approach, i.e., starting at the injection stage (Figure 2). Indeed, loss of signal was not expected at the injection stage into the UPLC-MS/MS (Over-Spike). Thus, the contribution of each phase of the extraction procedure to the loss of analyte was assessed by starting with the SPE columns and then working backwards to the PP.

Samples were spiked (Middle-Spike) with MC congeners prior to application to the SPE columns and the recoveries compared for three different SPE columns (Table 1 and Figure S1): Phenomenex StrataX (3 cc, 200 mg sorbent), Waters Oasis HLB (6 cc, 200 mg sorbent) and Waters Oasis PRiME HLB (6cc, 200 mg sorbent). Although in a preliminary experiment, Supelco Hybrid-SPE Phospholipid and Phenomenex C18-E columns were also tested, these did not provide reliable data (data not shown), and therefore were not considered any further. The StrataX column presented overall recoveries ranging between 63.4 and 112.7%, with the majority of congeners showing a recovery of 70%. The PRiME HLB column showed acceptable recoveries (80.6–114.7%) for congeners containing arginine residues (Table 1 and Figure S1), while a pronounced loss of non-arginated congeners was observed (recoveries: 57.9–78.3%). In contrast, the HLB column presented better recovery for all MC congeners tested (72.6–108.7%), with recoveries for the majority of congeners ranging between 85 and 100%. A two-way ANOVA analysis of the latter data suggested that the recoveries determined were not significantly different from a control recovery of 100 ± 10% obtained with MC congeners in MeOH solution (Over-Spike) applied to the UPLC-MS/MS without sample matrix and prior SPE column separation. Consequently, the HLB column was employed for all subsequent MC congener recovery and quantitation experiments. 

Liquid-Liquid Partitioning (LLP) experiments, carried out according to a previously published methodology [31] to remove hydrophobic matrix elements, were conducted to determine MC congener loss during LLP. For this, an MC congener mix was spiked into methanol (MeOH), which was subsequently overlaid with n-hexane, as in the extraction procedure (Figure 2). After 30 min, the n-hexane phase was separated from the MeOH phase; both fractions were dried and re-dissolved in MeOH, thus allowing for the determination of the distribution of MC congeners in the n-hexane and MeOH phases. Nearly 100% of each MC congener was found in the MeOH phase (Table 1, Figure S2), thus indicating that no, or at best, negligible, loss would occur during LLP due to the n-hexane clean-up.

Finally, the loss during protein precipitation with MeOH or acetonitrile (ACN) was investigated. Protein precipitates, spiked with an MC congener mix (Full-Spike), were extracted once (1×) or twice (2×) with the respective solvent (MeOH or ACN). The comparison of MeOH and ACN did not show a significant difference (2-way-ANOVA) in recovery (Table 2 and Figure S3). Neither was there a significant increase (2-way-ANOVA) in recovery when protein precipitates were extracted twice. Hence, all subsequent experiments were carried out with a single MeOH extraction of the protein precipitates. As neither the SPE nor the LLP resulted in a demonstrable loss of MC congeners (Table 1), the poor recovery observed when the complete extraction procedure was followed through (Table 2), i.e., including the initial protein precipitation step suggested that the protein precipitation itself was responsible for the high loss (poor recovery) of MC congeners observed. 

### 2.2. Use of Internal Standard

**Figure 3:**
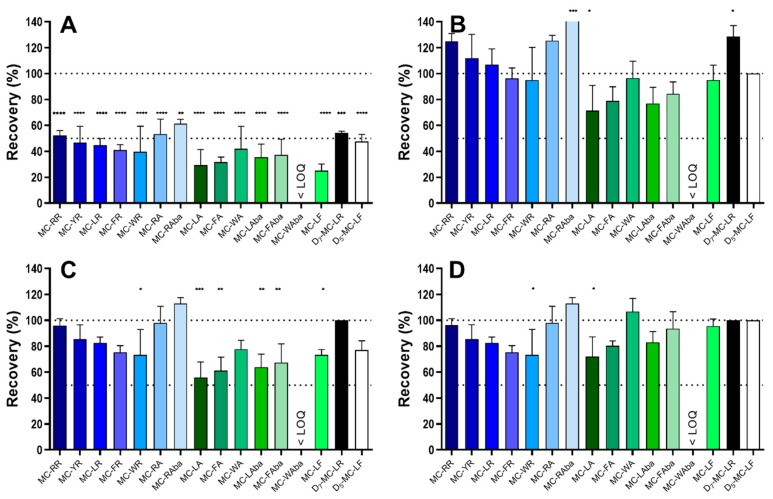
Quantification of MC in spiked human serum using D_5_-MC-LF and D_7_-MC-LR as internal standards (ISs). The same dataset was analysed using different IS for the quantification of the other MC congeners. (**A**) No internal standard specified, (**B**) D_5_-MC-LF as IS, (**C**) D_7_-MC-LR as IS, (**D**) D_7_-MC-LR as IS for all arginine-containing congeners and D_5_-MC-LF for non-arginine-containing congeners. n = 3, Two-way ANOVA with Bonferroni Post-test to test difference from 100 ± 20%. * *p* < 0.05, ** *p* < 0.01, *** *p* < 0.001, **** *p* < 0.0001.

D_5_-MC-LF and D_7_-MC-LR presented with similar total recovery as their respective non-deuterated analogues (*t*-test, *p* = 0.31 MC-LR vs. D_7_-MC-LR and *p* = 0.79 MC-LF vs. D_5_-MC-LF) (Table 2). Thus, use of these synthetic congeners could serve as an IS with which the recovery of individual MC congeners could be corrected. Hence, human serum was spiked with the MC congener mix, extracted with the established procedure (see above) and analysed using UPLC-MS/MS (Figure 3). Quantification of MC congeners without the IS for correction (Figure 3A) yielded the same result as obtained before, i.e., low recovery, thus demonstrating the robustness of the extraction and analytical procedure. When D_5_-MC-LF was used to quantify all other MC congeners (Figure 3B), recovery for non-arginine MCs was improved from around 40% to 80%; however, most arginine-containing congeners were overestimated. For example, when 10 µg/mL of D_5_-MC-LF were spiked to the sample, the resulting LC-MS/MS peak value was set as 100%, i.e., as if 100% recovery was achieved, and thus a correction factor for extraction loss was applied. The latter meaning that for the MC congeners to be determined the actual loss experienced during the extraction procedure was assumed to be identical to the loss observed for the IS. Accordingly, the actual values of the MC congeners to be determined were then multiplied with the correction factor for extraction loss as determined with the IS. In contrast, when D_7_-MC-LR (Figure 3C) was employed as IS, the recovery of arginine-containing congeners increased to 80–110%, whereas non-arginine-containing congeners were limited to a recovery of approximately 60%. The best results, i.e., recovery of most MC congeners of 100 ± 20%, were achieved when using a combination of D_5_-MC-LF and D_7_-MC-LR as IS (Figure 3D), whereby D_5_-MC-LF was employed for the correction of the recoveries of non-arginine-containing and D_7_-MC-LR for arginine-containing MC congeners, respectively. 

### 2.3. Method Validation

The established extraction procedure and UPLC-MS/MS detection method using deuterated MC ISs, developed for human serum (see above), was further tested by spiking murine serum and liver homogenate with the MC congener mix (Table 3). MC recoveries from mouse serum were similar to those observed for human serum regarding total recovery (Figure 3D vs. Table 3). In contrast, the recoveries from the more complex liver homogenate matrix provided mixed results. While, generally, arginine-containing MC congeners were recovered with an equally good recovery as those observed in murine serum, the recovery for some of the non-arginine-containing congeners, with the exception of MC-LF, ranged between ⅓ and ½ of the recoveries found for the respective MC congener in murine serum (Table 3). 

MC-RR, MC-YR and MC-LR were used as external standards, as recommended by the European Medicines Agency (EMA) [33], to determine the LOD, LOQ and linear range for the UPLC-MS/MS detection method used. LOD was defined as the minimal amount of the analyte which provided for a discernible peak, while LOQ was defined as the minimal amount of the analyte which produced a peak at least three times higher than the background (signal above noise) and has an accuracy <±20%. The LOD for each was slightly lower, i.e., 0.5 ng/mL for MC-RR and -YR and 0.1 ng/mL for MC-LR. The upper limit of the linear range might even be higher, but no higher concentrations were tested. The linear range was defined to lie between the LOQ and the highest standard concentration used in the experiment. Linear range for the three MC congeners was between 2 and 500 ng/mL, all with R^2^ > 0.99. 

To determine the precision of the UPLC-MS/MS detection method, intra-day and inter-day precision was determined. Identical samples were measured on two different days. Relative standard deviations (RSD) between 1.7 and 19.7% were observed for intra-day precision of day 1, although slightly higher similar RSDs (between 5.0 and 21.5%) were found on day 2. No obvious relationship between RSD and MC congener structure could be observed, i.e., precision for arginine-containing congeners and non-arginine-containing congeners was similar (Table 4). When means of the individual days were compared (inter-day precision), RSD ranged from 1.3% to 31.0%, again with no difference in variability among arginine- and non-arginine containing congeners (Table 4).

### 2.4. MC Levels in Exposed Mice

**Figure 4:**
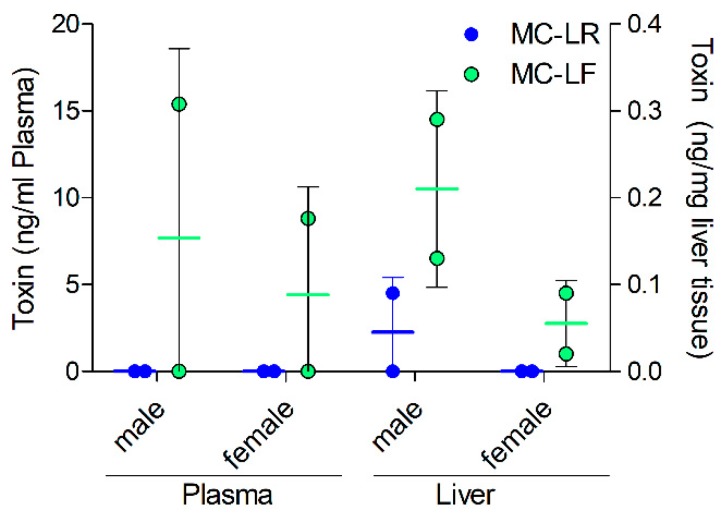
Analysis of MC levels in plasma and livers of exposed mice. Mice were injected with 100 µg/kg bw MC-LR or MC-LF. Values < LOD are shown as 0. Results are mean ± SD of two animals.

Liver and plasma samples of male and female Balb/c mice that were treated i.p. with 100 µg/kg bw MC-LR or MC-LF before sacrifice (24 h post-injection), were analysed using the extraction procedure and UPLC-MS/MS analytical method previously developed. While MC-LF was quantifiable in plasma and liver tissue for both male and female mice (Figure 4), MC-LR was detected only in the male liver tissue but not in female livers or the plasma samples of both sexes. It is to be noted that for both MC-LF and MC-LR, toxin levels in plasma and liver appeared to be lower in females than in males. The low number of animals available for this analysis prevented further investigation, i.e., corroboration of this observation. No MCs were observed in animals injected with 20 µg/kg bw of the toxins every second day for two weeks (data not shown).

## 3. Discussion

The method described here demonstrated the simultaneous detection of 14 MC congeners in biological samples (serum, plasma, liver homogenate) using synthetic deuterated MC internal standards. The complete method presented acceptable analyte recovery, low LOD and LOQ, and limited intra- and inter-day variation (Table 3 and Table 4). In principle, this method is also amenable to the simultaneous detection and quantification of >14 MC congeners, albeit this would come at the expense of decreased sensitivity for individual MC congeners, as per time unit more parent masses would need to be analysed. The latter could be partly alleviated by narrowing the specific windows of analysis, as described in the Methods section and shown in Table 5.

The quantification method presented here using two synthetic deuterated MCs as internal standards is, to the best of our knowledge, the first time this has been carried out for MCs. Previous studies have either employed MC-LR with an attached thiol group at the Mdha residue as an internal standard for the HPLC-MS analysis of MC-LR, -RR and -LA [27], or similarly thiolised MC-LR and -RR in conjunction with MALDI-MS [26]. The problem with the latter approach is that thiolised internal standards do not necessarily have the same behaviour during extraction and analysis as the parent compounds. Moreover, these ISs are based on arginine-containing MC congeners, thus introducing a bias when measuring non-arginated MC congeners. In contrast, the ISs used here (D_5_-MC-LF and D_7_-MC-LR) represent two major classes of MC, i.e., arginated and non-arginated congeners, and more importantly do not incorporate structural changes of the IS that would change behaviour during extraction and analysis. Other studies have suggested that heavy atoms are the best choice for labelling, because deuterium may change the analytical properties of the standards, e.g., retention time and hydrophobicity, possibly leading to altered recovery [34,35]. However, this was not observed during the present study with recovery (Supplementary Figures S1 and S3) and retention times being indistinguishable from those observed for the respective naturally occurring (non-deuterated) MC congeners.

Although the new procedure for quantifying MC congeners in biological tissue samples is promising, it must be noted that the analysis is still restricted to free and unconjugated MC congeners, as it uses the parent ion mass of each congener analysed. It has been suggested that free MC only comprises a minor portion of the total MC load after exposure [2,36], so one would assume that the actual MC load is severely underestimated [19]. Indeed, MC-LR was nearly undetectable in liver and plasma of mice injected i.p., with 100 µg/kg bw of MC-LR (Figure 4). While the lack of MC-LR detection in plasma samples could be interpreted to be the result of rapid uptake via the mOatp1b2 [37,38], lack of MC-LR detection in the liver samples could suggest either covalent protein binding or rapid excretion, and thus elimination from the liver. Indeed, as MC-LF was detectable in the liver samples and the covalent interaction of MC-LF and MC-LR are assumed to be comparable, it is currently undiscernible whether the differences of liver tissue levels stem from differences in kinetics (uptake and excretion of MC congeners), dynamics (covalent binding) or indeed difference in extraction of free and protein bound MC. Recently, methods have been developed that are capable of cleaving the thiol bond formed during conjugation and thus release free MC using basic conditions [39,40,41]. Moreover, the regioselective cleavage of the thiol bond in combination with the application of our IS to future in vivo or in vitro experiments would allow to quantify the amount of free, conjugated and protein-bound single MC congeners or MC congener mixtures. 

ESI-MS/MS methods are known to be influenced by matrix components left in the sample subsequent to extraction (matrix-effects) [42]. Although ISs were employed, the matrix still influenced the signal of the individual MC congeners. It is interesting to note that the liver matrix appeared to influence the recovery of the non-arginated MC congeners MC-LA, MC-FA, MC-WA, MC-LAba, MC-FAba, and MC-WAba (34.1–64.5% recovery) more profoundly than other non-arginated (MC-LF, 90.6%) or arginated MC congeners (78.7–120.9% recovery) (Table 3).

Ideally, method validation would be carried out with human and murine serum and plasma, albeit no marked difference was observed between the application of the method on human or mouse serum (Figure 3D vs. Table 3). Nevertheless, previous studies suggested stronger binding of MC congeners to human than to murine or other mammalian albumin [43,44], thus the recoveries obtained for 14 MC congeners in human serum (Figure 3) with those observed in murine plasma (Figure 4, Table 3) would suggest that the differences in albumin binding had an impact. In consequence, method validation would have to be carried out for every sample type prior to broad data analysis. 

As method validation was carried out in murine plasma, more trust is placed in the results of the plasma analyses of the mouse experiments in vivo than in those in the liver homogenates. Irrespective of the latter, MC could not be detected in plasma or liver tissue of mice which were injected with 20 µg/kg bw MC-LR or MC-LF every second day for 2 weeks, while mice exposed to 100 µg/kg bw MC-LR and LF showed detectable MC levels both in plasma and liver tissue (Figure 4). Studies in humans [45,46] observed serum levels of around 0.2–0.6 ng/mL in humans consuming contaminated food items and drinking water. Here, we could detect MCs in mouse plasma at around 10–15 ng/mL after injection of a single dose 100 µg/kg bw. This shows, that the detection limit is biologically relevant and that the range in which the presented method is linear fits to actually observed levels. MCs are known to be efficiently conjugated to glutathione [47] and are subsequently rapidly excreted [48,49], most likely via the bile into the small intestine. Thus, it may not be surprising that application of small concentrations of MCs every two days for 2 weeks resulted in non-detectable free MC in the plasma and liver of exposed mice. If a regioselective cleavage of the thiol bond had been applied to the plasma and liver homogenates prior to the MC extraction procedure, protein-bound MC would most likely have been detected. Indeed, a recent study in pigs showed that oral consumption via drinking water over several weeks at low MC-LR doses (2 µg/kg bw) did not lead to any detectable free microcystins in plasma and livers, similar to our study. However protein-bound MC-LR could be detected in the livers at around 20 ng/mg using Lemieux oxidation [50]. Thus, the implementation of regioselective cleavage to assess the fraction of bound MCs is essential, especially as it was found that around 85% of administered MC-LR amounts are found in a bound state in fish tissue [19].

The fact that MC-LF was readily detectable in plasma and liver homogenates of male and female mice, whereas this was not the case for MC-LR, could suggest that either the murine Oatp1b2 expressed in the liver is more proficient in taking up MC-LR than MC-LF or that murine hepatocytes conjugate and excrete MC-LR faster than MC-LF. Differences in binding affinity of MC-LF and-LR for the murine organic anion polypeptide transporter mOatp1b2 have been observed [37,38]. Interestingly, females tended to show lower detectable amounts of MC-LF in both matrices than males, an observation that could stem from a sex-dependent expression levels of Oatp1b2 which is hormonally regulated, as observed for other murine Oatps [44,51]. However, the latter findings need to be taken with caution, as only two replicates were used. Nevertheless, similar quantities of MC-LR, based on wet weight, were found in livers of the mice in our study when compared to the livers of rats used in the study of Wang, Xie, Chen and Liang [48]. 

In summary, we established a procedure capable of simultaneously quantifying 14 MC congeners in plasma/serum and liver tissue samples. For the first time, de novo synthesised deuterated MC internal standards were used for quantification and resulted in a highly improved recovery of MC congeners. The method was validated using a matrix of plasma and was applied to the detection of administered MC in mouse plasma and liver samples. In the future, the regioselective cleavage of the thiol bond in combination with the application of our deuterated ISs should allow for the quantification of free, conjugated (e.g., glutathione) and protein-bound single MC congeners or MC congener mixtures.

## 4. Materials and Methods 

### 4.1. Materials

Human serum (off the clot) was obtained from Biochrom (Berlin, Germany) or from the New Zealand Blood Service. Water was deionised water with 18.2 MΩ (Milli-Q). Formic acid (Merck, Darmstadt, Germany), acetonitrile (Carl Roth, Karlsruhe, Germany) and solvents were of MS grade. Analytical standards for MC-RR, MC-LR and MC-YR were from DHI LAB products (Hørsholm, Denmark). SPE columns were either from Waters (Oasis HLB and PRiME HLB, Eschborn, Germany) or from Phenomenex (StrataX, Auckland, New Zealand). They were used with a vacuum manifold by Macherey-Nagel (Düren, Germany).

For UPLC-MS/MS analytics, an internal standard was used which comprised synthetic, deuterated MC-LR and MC-LF. The synthesis of D_5_-MC-LF (containing phenylalanine with a deuterated phenyl group (five D-atoms) in position 4) was published previously [52]. D_7_-MC-LR was synthesised in analogy to the published synthesis of D_5_-MC-LF employing leucine deuterated in the side chain (seven D-atoms) at position 2. These two standards were either used for spiking mouse plasma and liver samples (see below), or supplementing (100 ng/mL each) a mixture of MCs (MC-mix) extracted from *Microcystis CAWBG11* cells as published previously [28,32]. The extract contained multiple microcystin congeners, among them MC-RR, -YR, -LR, -FR, - WR, -RA, -RAba, -LA, -FA, -WA, -LAba, -FAba, -WAba and was additionally supplemented with each 100 ng/mL MC-LF. The so-produced stock solution (MC congener mix) was used for spiking into samples at a 1:10 ratio to establish the extraction and detection method. MC concentrations ranged between 94 ng/mL (MC-WAba) and 2768 ng/mL (MC-LR) in the stock solution.

### 4.2. Sample Generation for the Establishment and Validation

All liquid samples were handled in glass LC-vials to reduce loss due to adsorption, as previously recommended [31,32].

Serum/plasma samples for method establishment (0.5 mL) were spiked with MC congener mix stock at a 1:10 ratio (MC mix:serum, *vol/vol*). A 1:10 dilution of the MC mix in methanol, serving as control for every experiment, was analysed without further treatment (Recovery control). Spiking was performed at different steps during the procedure (Figure 2); before extraction (Full-Spike), after extraction but before UPLC-MS/MS analysis (Over-Spike) or directly before the SPE step (Middle-Spike). These samples were used for generating the data in Table 1 and Table 2, and Figure 3.

Additional establishment and validation experiments were performed with plasma and livers of non-treated Balb/c mice obtained from the animal facility of the University of Konstanz. Serum/plasma (0.25 mL) was used as is, while liver samples were homogenised prior to spiking. For homogenisation, livers were thawed on ice, pieces taken, weighed, and 200% ice-cold RIPA buffer was added (e.g., 125 mg liver + 250 µL RIPA). The sample was then homogenised using an electric drill with attached pestle while on ice. The final liver homogenate (250 µL) was used for spiking and subsequent toxin extraction, resulting in 83.3 mg of tissue used for one sample. Spiking was carried out with the MC-congener mix at a ratio of 1:10 (*vol*/*vol*). These samples were used during the method validation (Table 3).

### 4.3. Extraction Method for MC from Blood and Liver Tissue Samples

Samples (250 µL liver homogenates or plasma/serum) were transferred to glass reaction tubes and subjected to protein precipitation by adding three volumes 100% MeOH. Precipitates were centrifuged for 40 min at 4 °C with 3023 rcf. Subsequently, supernatants were transferred to clean glass tubes. Removal of lipophilic compounds from the supernatant was conducted using n-hexane (4 mL). Samples were vortexed, phases were allowed to separate for 30 min before the n-hexane was removed and discarded. The sample was diluted with MilliQ (52 volumes) to reduce the organic solvent content below 10% (*v*/*v*) in the final sample. Afterwards, samples were subjected to solid-phase extraction (SPE) clean-up using Waters Oasis HLB (6 cc, 200 mg sorbent). Columns were connected to a vacuum manifold, activated with 100% MeOH (5 mL) and equilibrated with 10% MeOH (5 mL). Then the sample was loaded, washed with MilliQ (7 mL) and 20% MeOH (7 mL) before elution with 80% MeOH (5 mL). All SPE steps were performed at a maximum vacuum of 20 kPa. SPE eluents were dried with a SpeedVac before reconstitution in 0.25 or 0.5 mL pure MeOH and stored at −20 °C until analysis. Samples of the animal study had to be concentrated further. In the latter case, samples were again dried using the SpeedVac and reconstituted in 12.5 µL pure MeOH.

### 4.4. UPLC-MS/MS Analysis

Analyses were performed on a Waters Acquity H-class liquid chromatograph equipped with an Acquity BEH C18 column (1.6 µm, 2.1 × 50 mm) with a corresponding guard column kept at 40 °C, coupled to a Waters XEVO TQ-S mass spectrometer. The used solvents were 10% ACN (solvent A) and 90% ACN (solvent B), both containing 100 mM CH_2_O_2_ and 6 mM NH_3_ with a total flow of 0.4 mL/min. The used gradient started with 25% B, held for 30 s at 25% B before increasing to 45% B over additional 30 s. Over the following 180 s amount of solvent B increased to 60% before being raised to 99% over 12 s where it was held for additional 30 s. Reequilibration back to 25% B was done over 78 s, where it was held for another 60 s before the next sample injection. Injection volume was 5 µL. Compounds were ionised using a capillary voltage of 3 kV and a nebuliser pressure of 7.0 bar. Dissolution was achieved using a nitrogen flow of 1000 L/h at 500 °C. Analysis of the congeners was divided into five analysis windows, to reduce the number of parallel analysed congeners in order to maximise the time spent scanning for each individual compound. Analysis parameters for all MC congeners are shown in Table 5.

MC-RR, MC-YR and MC-LR were used as external standards for each analytical run. Standards were used at three final concentration levels: 2, 10 and 100 ng/mL and employed for establishing a linear regression of the signal response with the injected amount. In the first experiments, these standards were used in MeOH, during method validation standards were used in blank extracted matrix. The external standard MC congener mix also contained MC-LF, which was not from a source classified as reference material. Therefore, it was not used to quantify any MC levels, but only as a point of comparison to the other standards during the validation. D_7_-MC-LR as IS for all arginine-containing congeners and D_5_-MC-LF for non-arginine-containing congeners were either spiked into samples individually before extraction (animal experiment) or were part of the MC-mix used for establishment and validation.

### 4.5. Animal Samples

Balb/c mice, obtained from Janvier (Le Genest-Saint-Isle, France), were injected i.p. with either MC-LR, MC-LF (20 µg/kg bw) or vehicle (water) every second day for 14 days, before sacrifice of the animals and organ harvest. Additional mice were injected once with 100 µg/kg bw MC-LF or MC-LR. Mice were sacrificed 24 h after last injection. Blood was taken in heparinised tubes, centrifuged and the remaining plasma was snap-frozen in liquid nitrogen. Liver tissue was also snap-frozen in liquid nitrogen. Samples were stored at −80 °C until further preparation. Mice were housed in plastic cages, in an air-conditioned room (20–23 °C) under a 12/12 h light/dark cycle with free access to rodent pellets and tap water. The animals were acclimatised at least 7 days prior to experimentation. Animal handling, exposure and organ removal was performed in the laboratory of ANSES (Agence nationale de sécurité sanitaire de l’alimentation, de l’environnement et du travail) Fougères in 2008 according to approved protocols by the Institute’s ethical committee on animal experimentation.

For spiking experiments, plasma samples were used as is, while livers were homogenised in analogy to the validation samples (see above). Every sample was spiked with 10–50 ng/mL D_7_-MC-LR and D_5_-MC-LF, as IS, and samples stored at −20 °C until extraction.

### 4.6. Data Analyses and Statistics

LC-MS/MS data was analysed using Targetlynx 4.1 integrated into MassLynx 4.1 (Waters). Data handling was performed with Microsoft Excel, while GraphPad Prism 5 was used for statistical analysis and data visualisation.

## Figures and Tables

**Table 1:** Congener dependent recovery during SPE and LLP steps of the extraction.

	Solid Phase Extraction (SPE)						Liquid-Liquid Partitioning (LLP)			
	StrataX		HLB		PRiME HLB		% in MeOH		% in n-Hexane	
	Mean	SD	Mean	SD	Mean	SD	Mean	SD	Mean	SD
MC-RR	63.4	2.3	90.4	6.0	100.9	5.8	89.0	9.9	0.6	0.0
MC-YR	112.7	2.2	81.2	5.6	80.6	9.8	96.4	4.6	1.0	0.1
MC-LR	79.5	2.5	95.1	5.3	97.8	7.4	98.5	4.8	0.5	0.0
MC-FR	66.8	12.4	97.6	6.9	107.2	7.2	97.8	7.0	0.6	0.0
MC-WR	74.9	2.2	98.5	6.6	114.7	7.9	97.8	8.5	0.5	0.0
MC-RA	87.7	5.5	104.8	6.6	98.0	4.3	97.3	4.2	0.6	0.1
MC-Raba	105.9	0.0	108.7	10.9	102.3	7.4	100.0	6.2	0.0	0.0
MC-LA	76.0	2.1	72.6	7.1	57.9	5.6	93.6	2.8	0.8	0.0
MC-FA	67.9	3.3	81.6	8.5	62.2	3.7	93.1	4.7	1.0	0.1
MC-WA	75.0	1.4	87.3	4.6	70.2	5.6	95.8	1.8	3.1	1.0
MC-LAba	69.2	5.8	86.5	7.1	68.1	4.0	n.d.	n.d.	n.d.	n.d.
MC-FAba	68.4	4.6	86.9	8.5	78.3	2.5	n.d.	n.d.	n.d.	n.d.
MC-WAba	71.9	3.0	n.d.	n.d.	n.d.	n.d.	n.d.	n.d.	n.d.	n.d.
MC-LF	n.d.	n.d.	n.d.	n.d.	77.4	4.7	n.d.	n.d.	n.d.	n.d.
D_7_-MC-LR	n.d.	n.d.	97.8	6.6	92.6	5.9	n.d.	n.d.	n.d.	n.d.
D_5_-MC-LF	n.d.	n.d.	94.8	6.7	79.1	3.9	n.d.	n.d.	n.d.	n.d.

For the determination of the loss of the SPE procedure, MC congeners in MeOH solution were spiked to the sample after protein precipitation and liquid-liquid partitioning, but before the SPE procedure (Middle-Spike). Liquid-liquid partitioning was performed with MC-spiked MeOH, which was topped with hexane. n.d.: not determined in that particular experiment. n = 3. SD = standard deviation.

**Table 2:** Total recovery of the extraction using different protein precipitation (PP) procedures.

	Methanol				Acetonitrile			
	1×		2×		1×		2×	
	Mean	SEM	Mean	SEM	Mean	SEM	Mean	SEM
MC-RR	43.2	2.1	52.3	5.0	44.6	2.6	47.1	2.8
MC-YR	35.1	2.2	47.4	7.1	43.8	4.2	47.2	3.0
MC-LR	43.8	2.3	49.4	5.2	45.9	2.8	46.7	2.6
MC-FR	44.3	2.3	56.3	6.0	50.8	2.9	52.1	3.5
MC-WR	41.0	3.0	54.0	5.6	45.6	3.3	48.0	3.2
MC-RA	50.9	2.2	62.0	7.5	54.8	2.5	55.7	2.7
MC-Raba	52.9	3.7	62.5	7.3	54.5	2.3	59.9	2.4
MC-LA	49.2	2.4	35.2	3.5	54.2	2.7	47.7	3.6
MC-FA	47.5	2.3	41.8	4.4	55.2	2.4	50.7	1.8
MC-WA	45.7	2.6	49.0	3.8	48.7	2.5	50.2	3.7
MC-LAba	49.7	2.8	47.0	6.0	57.7	3.0	56.6	3.3
MC-FAba	50.9	2.2	52.9	6.0	59.4	2.5	64.7	3.6
MC-WAba	0.0	0.0	0.0	0.0	0.0	0.0	0.0	0.0
MC-LF	59.2	5.3	61.1	5.8	69.0	6.4	64.3	6.2
D_7_-MC-LR	48.0	2.2	51.9	4.1	51.0	2.6	51.0	2.9
D_5_-MC-LF	61.0	2.5	67.4	6.0	70.0	3.1	71.6	3.3

n.d.: not determined in that particular experiment. n = 2 (MeOH) and 3 (ACN). SEM = standard error of the mean.

**Table 3:** Validation parameters of the established methods.

	Mouse Recovery (% of Expected Result)		LOD (ng/mL)	LOQ (ng/mL)	Linear Range (ng/mL)
Congener	Mouse Serum	Mouse Liver	Mouse Serum	Mouse Serum	Mouse Serum
MC-RR	74.2%	78.7%	0.5	2	2 - 500
MC-YR	88.5%	82.9%	0.5	2	2 - 500
MC-LR	113.1%	81.2%	0.1	2	2 - 500
MC-FR	124.3%	86.3%	-	-	-
MC-WR	108.6%	94.5%	-	-	-
MC-RA	111.2%	120.9%	-	-	-
MC-RAba	106.4%	109.6%	-	-	-
MC-LA	86.1%	64.5%	-	-	-
MC-FA	100.0%	43.4%	-	-	-
MC-WA	96.8%	34.1%	-	-	-
MC-LAba	94.3%	42.2%	-	-	-
MC-FAba	97.4%	54.4%	-	-	-
MC-WAba	89.8%	<LOQ	-	-	-
MC-LF	83.5%	90.6%	(0.5)	(2)	(2 - 500)
D_7_-MC-LR	100.0%	100.0%	-	-	-
D_5_-MC-LF	100.0%	100.0%	-	-	

MC-RR, MC-YR and MC-LR were used as external standards. MC-LF was also part of the external standard mix, but was not used to quantify any of the other MC congeners as it was no certified reference material but served as reference for more hydrophobic congeners. D_7_-MC-LR and D_7_-MC-LR were used as internal standards in every sample quantified. Recovery was calculated using the external standards in MeOH. Therefore, the recovery as calculated here shows the influence of the matrix. MC congener mix spiked mouse plasma and liver homogenates from untreated male animals were used for this experiment. Linear regressions for the linearity determination all showed R^2^ > 0.99.

**Table 4:** Intra-day and inter-day precision.

	Day 1			Day 2			Both Days
	Mean (ng/mL)	SD (ng/mL)	RSD (%)	Mean (ng/mL)	SD (ng/mL)	RSD (%)	RSD (%)
MC-RR	**193.0**	**10.0**	**5.2**	**208.3**	**44.7**	**21.5**	**5.4**
MC-YR	**31.1**	**3.4**	**10.8**	**32.7**	**6.5**	**19.9**	**3.5**
MC-LR	**502.7**	**22.8**	**4.5**	**682.9**	**44.0**	**6.4**	**21.5**
MC-FR	271.9	14.5	5.3	383.0	43.5	11.4	24.0
MC-WR	81.6	16.1	19.7	127.3	16.9	13.3	31.0
MC-RA	73.5	9.4	12.8	75.8	13.5	17.8	2.2
MC-RAba	21.1	1.0	4.5	22.6	2.7	12.1	5.0
MC-LA	112.6	17.9	15.9	102.9	8.6	8.3	6.4
MC-FA	145.5	2.4	1.7	119.6	6.0	5.0	13.8
MC-WA	46.3	3.1	6.7	38.0	5.5	14.6	14.0
MC-LAba	89.0	10.2	11.5	87.4	6.5	7.4	1.3
MC-FAba	49.7	8.1	16.2	39.1	3.1	8.0	16.9
MC-WAba	<LOQ	n.d.	n.d.	< LOQ	n.d.	n.d.	n.d.
MC-LF	<LOQ	n.d.	n.d.	< LOQ	n.d.	n.d.	n.d.
D7-LR	10.0	0.0	0.0	10.0	0.0	0.0	0.0
D5-LF	10.0	0.0	0.0	10.0	0.0	0.0	0.0

Samples (n = 3) were measured on day 1. RSD is a measure for intraday precision. Identical samples were also measured on day 2 with slightly different RSD. The last column shows the RSD when the means of day 1 and day 2 are used.

**Table 5:** MS Parameters.

Congener	Analysis Window (min)	Parent Mass (*m*/*z*)	Daughter Mass (*m*/*z*)	Dwell Time (s)	Cone Voltage (V)	Collision Energy (V)	External Standard	Internal Standard
MC-RR	0.3–1.6	519.7	135.1	0.027	40	27	MC-RR	D_7_-MC-LR
MC-YR	0.3–1.6	1045.6	135.1	0.027	40	70	MC-YR	D_7_-MC-LR
MC-LR	0.3–1.6	995.6	135.1	0.027	40	65	MC-LR	D_7_-MC-LR
MC-FR	0.3–1.6	1029.6	135.1	0.027	40	65	MC-LR	D_7_-MC-LR
MC-WR	0.3–1.6	1086.6	135.1	0.027	40	65	MC-LR	D_7_-MC-LR
MC-RA	1.3–2.5	953.6	135.1	0.024	40	65	MC-LR	D_7_-MC-LR
MC-Raba	1.3–2.5	967.6	135.1	0.024	40	65	MC-LR	D_7_-MC-LR
MC-LA	1.3–2.5	910.6	135.1	0.024	40	65	MC-LR	D_7_-MC-LF
MC-FA	1.3–2.5	944.6	135.1	0.024	40	65	MC-LR	D_7_-MC-LF
MC-WA	1.3–2.5	983.6	135.1	0.024	40	65	MC-LR	D_5_-MC-LF
MC-Laba	1.7–3.5	924.6	135.1	0.024	40	65	MC-LR	D_5_-MC-LF
MC-Faba	1.7–3.5	958.6	135.1	0.024	40	65	MC-LR	D_5_-MC-LF
MC-WAba	1.7–3.5	997.6	135.1	0.024	40	65	MC-LR	D_5_-MC-LF
MC-LF	1.7–3.5	986.6	135.1	0.024	40	65	MC-LR	D_5_-MC-LF
D_7_-MC-LR	1.0–1.8	1002.7	135.1	0.024	40	65	MC-LR	-
D_5_-MC-LF	2.3–3.5	991.6	135.1	0.024	40	65	MC-LR	-

The columns for internal and external standard denominate the congeners, which are used for the quantification of the respective conger during the UPLC-MS/MS analysis. Dwell times differ as the individual analytical windows do not contain the same numbers of congeners.

## Supplementary Materials

The following are available online at https://www.mdpi.com/2072-6651/11/7/388/s1, Figure S1: Column comparison using a middle-spike in serum, Figure S2: Distribution of microcystins in methanol and hexane fractions, Figure S3: Use of acetonitrile and methanol for protein precipitation.
