# Supplementary material for: Simultaneous Detection of 14 Microcystin Congeners from Tissue Samples Using UPLC- ESI-MS/MS and Two Different Deuterated Synthetic Microcystins as Internal Standards
Source: Toxins (Basel). 2019 Jul 2;11(7):388. doi: 10.3390/toxins11070388 (PMC6669509; doi:10.3390/toxins11070388)
Supplement: Supplementary file 1 [file toxins-11-00388-s001.pdf]

Supplementary Materials: Simultaneous Detection of 14 Microcystin Congeners from Tissue Samples Using UPLC- ESI-MS/MS and Two Different Deuterated Synthetic Microcystins as Internal Standards

Stefan Altaner, Jonathan Puddick, Valerie Fessard, Daniel Feurstein, Ivan Zemskov, Valentin Wittmann and Daniel R. Dietrich

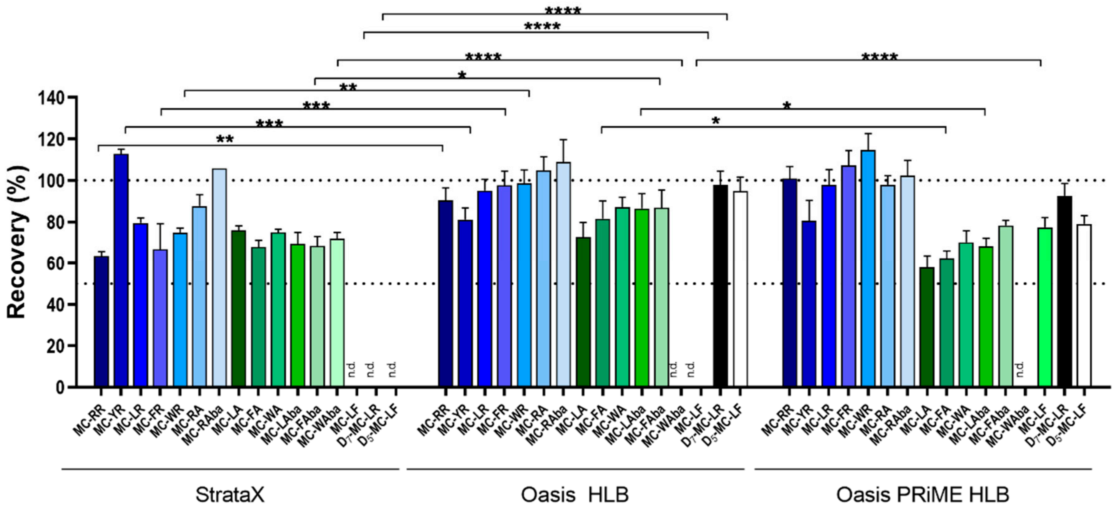

Figure S1. Column comparison using a middle-spike in serum. Blank serum was extracted with the PP and LLP, then spiked with QC mix before SPE. Interfering residue seems lowest on HLB columns, higher with StrataX and Prime HLB. n.d.: not determined in that specific experiment. Here, human plasma was used.

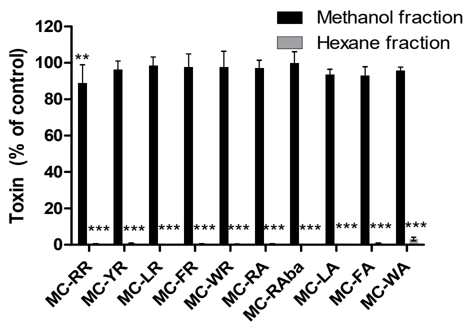

Figure S2. Distribution of microcystins in methanol and hexane fractions. Microcystins were spiked into methanol, hexane phase added, mixed, phases allowed to separate and, both phases dried. Microcystins were then reconstituted in MeOH and measured. Two-way ANOVA with Bonferroni posttest was used to calculate significant difference from "artificial" 100% control. ** $p < 0.01$; *** $p < 0.001$.

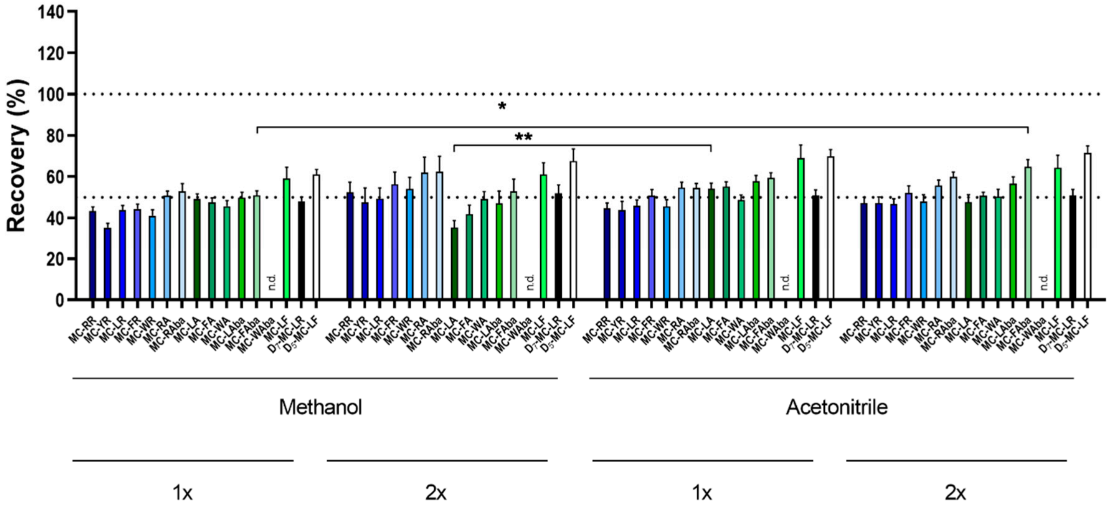

Figure S3. Use of acetonitrile **a**nd methanol for protein precipitation. Serum was spiked before extraction. The protein precipitation step was either performed with methanol or acetonitrile for one or two times, SPE was performed using the Oasis HLB columns. No difference in recovery was observed, therefore the easiest option was taken for further experiments: single extraction with methanol. Human serum (off the clot was used).
